# Poor Glycemic Control Increases Dental Risk in a Sri Lankan Population

**DOI:** 10.3390/healthcare12030358

**Published:** 2024-01-31

**Authors:** Larissa Steigmann, Sejal Gunaratnam, William V. Giannobile, Monica Van Til, Stephanie Daignault-Newton, William H. Herman, Naresh Gunaratnam, Prasad Katulanda, Aruna V. Sarma

**Affiliations:** 1 Division of Periodontology, Department of Oral Medicine, Infection, and Immunity, Harvard School of Dental Medicine, Boston, MA 02115, USA; 2 Department of Internal Medicine, University of Michigan School of Medicine, Ann Arbor, MI 48109, USA; 3 Department of Urology, University of Michigan School of Medicine, Ann Arbor, MI 48109, USA; 4 Huron Gastroenterology and Associates, Ypsilanti, MI 48197, USA; 5 Faculty of Medicine, University of Colombo, Colombo 00300, Sri Lanka

**Keywords:** diabetes mellitus, oral health outcomes, periodontitis, tooth loss, preventative interdisciplinary care, dental risk assessment

## Abstract

**Introduction:** The aim of our study was to investigate the impact of diabetes-related factors on the dental disease outcomes of diabetes patients in Trincomalee, Sri Lanka. **Materials and Methods:** Dental data were collected from 80 type-2-diabetic individuals. A dental risk score was calculated based on the frequency of dental outcomes observed and categorized as low risk (≤3 dental outcomes) and high risk (>3 dental outcomes). **Results:** In this cohort of men and women with type 2 diabetes, there was a high frequency of periodontal related outcomes, including missing teeth (70%), gingival recessions (40%), tooth mobility (41%), and bleeding (20%). Thirty-nine (39%) of participants had high dental risk, while forty-nine (61%) had low risk. **Conclusions**: After controlling for age, participants with higher capillary blood glucose levels had 3-fold greater odds of a high dental risk score (OR = 2.93, 95%CI = 1.13, 7.61). We found that poor glycemic control indicated by elevated capillary blood glucose was associated with increased dental risk.

## 1. Introduction 

The global prevalence of diabetes mellitus has seen a rapid increase, making it one of the leading causes of morbidity and mortality worldwide [1]. This surge in global morbidity and mortality, particularly in low- and middle-income countries (LMICs) [2], is exemplified by Sri Lanka, an island nation just south of India. Sri Lanka serves as a microcosm reflecting the challenges associated with diabetes in LMICs, encompassing its rising prevalence due to a growing and aging population and the difficulties in managing the increasing burden and related complications [3]. The overall prevalence of diabetes in Sri Lanka for adults aged 20 to 79 during this time was 8.7%, reaching rates as high as 16.5% in urban areas [4]. Periodontal disease is a chronic inflammatory condition characterized by the destruction of the periodontal tissues and resulting in the loss of connective tissue attachment, the loss of alveolar bone, and the formation of pathological pockets around the diseased teeth [5]. Periodontal disease continues to be a global public health problem and is the leading cause of tooth loss in adults [6]. Diabetes mellitus is an established risk factor for periodontitis, and active periodontitis has also been shown to impair glycemic control [7]. The literature supports this two-way interaction between diabetes and periodontitis, and periodontitis has even been reported to be “*the sixth complication of diabetes mellitus*” [8]. The association between diabetes and periodontal disease has been explored extensively and, while it is generally accepted that periodontal disease is more prevalent and severe in persons with diabetes [9], the data on the prevalence of periodontitis among those individuals with diabetes in Sri Lanka are limited to one study in a large urban population in Colombo, Sri Lanka [10]. Hence, the objective of this study is to determine the burden of periodontal outcomes and to investigate the impact of diabetes-related factors on these outcomes in a cohort of diabetic patients in Trincomalee, Sri Lanka. 

## 2. Materials and Methods

### 2.1. Study Population

Study participants included 80 individuals who had a confirmed history of type 2 diabetes and not taking insulin from three hospitals in Trincomalee, Sri Lanka; (1) Trincomalee General Hospital, (2) Selvanayagapuram Hospital, and (3) Sampaltheevu Hospital, Trincomalee, Sri Lanka; and who underwent an oral health exam. The study was approved by the University of Michigan (USA) Institutional Review Board (HUM00234099), as well as the Sri Lanka Eastern Province Health Minister. Informed consent was obtained from all participants included in the study. 

### 2.2. Data Collection and Analysis

Each study participant provided information on their date of birth, gender, smoking history, duration of diabetes, current diabetes treatment regimen, and recent hypoglycemic symptoms (such as nervousness, diaphoresis, tremors, and loss of consciousness). Participants also reported the time since their last oral intake, excluding water. Physical measurements, including height, weight, blood pressure, and capillary blood specimens, were collected. Capillary blood glucose (CBG) levels were measured using a CONTOUR^®^NEXT Blood Glucose Monitoring System (Ascensia Diabetes Care, Basel, Switzerland), and glycated hemoglobin (HbA1c) levels were measured using a DCA Vantage^®^ Analyzer (Siemens Medical Solutions USA, Malvern, PA, USA), imported from the USA due to the local scarcity of HbA1c testing resources. Both instruments underwent quality control procedures in accordance with the manufacturers’ specifications. Oral health exams were conducted on participants by certified dental providers from the USA and included the assessment of the presence of any of the following nine dental outcomes: (1) bleeding on probing, (2) gingival recession, (3) dental caries, (4) dental or periodontal abscess, (5) tooth mobility, (6) tooth fracture, (7) tooth loss, (8) pre-cancer, and (9) oral cancer. A dental score was calculated for each participant based on the frequency of dental outcomes observed and categorized as low risk (≤3), and high risk (>3). Demographic, clinical, and diabetes characteristics were examined and compared by dental score strata using *t*-tests for continuous variables, the Jonckheere–Terpstra trend test for ordinal variables, chi-square tests for nominal categorical variables, and Fisher’s exact tests, as appropriate. To test for the associations between diabetes characteristics and dental score, Poisson regression models for dental score were fit with the HbA1C, capillary blood glucose, and hypoglycemia in separate models as covariates, adjusted for age. All analyses were conducted using SAS 9.4 (SAS Institute, Cary, NC, USA).

## 3. Results

The prevalence of the individual dental outcomes assessed and associated dental risk scores are presented in Table 1. The highest-frequency dental outcomes were missing teeth (70%, *n* = 56), followed by dental caries (59%, *n* = 47), tooth fractures (49%, *n* = 39), and mobile teeth (41%, *n* = 33). Only two participants had affirmed oral cancer (2.5%). The frequency of dental outcomes ranged from 0 to 7 in the study population, with 31 (39%) identified as low risk (≤3), and 49 (61%) as high risk (>3) based on the dental risk score.

Demographic, clinical, and diabetes characteristics are presented, overall and stratified by dental risk scores, in Table 2. The median (IQR) age overall was 57 (50–64), with most participants being female (78%) and non-smokers (99%). Approximately half of the cohort were overweight/obese (44%). Less than half of the participants had a systolic blood pressure (SBP) of ≥130 mmHG (45%), and exactly half of them had an increased diastolic blood pressure (DBP) of ≥80 mmHg (50%). The majority of the participants reported the usage of diabetes medication (96%). A greater proportion of the participants were uncontrolled with an HbA1c > 7% (79%) and were at increased dental risk, albeit not significant for dental risk scores (*p*-value = 0.1). When adjusted for age, those with increased capillary blood glucose levels had significantly greater odds of being at dental risk (OR = 2.93, 95% CI = 1.13, 7.61). 

## 4. Discussion

We described the distribution of dental outcomes and associated dental risk scores and the impact of diabetes on dental risk score among a low-and middle-income country’s type-2-diabetic population. We found a significant burden of dental outcomes in this population and observed increased dental risk scores for participants with higher capillary blood glucose levels. 

The burden of the periodontal outcomes was significant in this cohort (Table 1). It is higher than previously described studies of individuals with type 2 diabetes in Sri Lanka and India [10,11]. The rates across the individual studies varied, likely, in part, due to heterogeneity in the diagnostic criteria and assessment methods used to identify periodontal disease. Our observation of higher risk of periodontal outcomes may also be likely related to the older mean age of the current cohort relative to those previously published, as well as a significantly high proportion of men and women with poor glycemic control. In more urban surroundings in Sri Lanka (Colombus district), a trend of increase in periodontal burden, including a rise in severity and prevalence, has been found in more recent studies [12].

Glycemic control is known to play a significant role in the development of periodontal complications [10]. Consistent with findings by Preshaw et al. [10], we observed that individuals experienced a significant increase in the risk of periodontal related dental outcomes with increasing capillary blood glucose levels after adjustment for age. Similarly, US adults with poorly controlled diabetes in the National Health and Nutrition Examination Survey (NHANES) III had a nearly 3-fold higher rate of severe periodontitis than those without diabetes after controlling for age, sex, ethnicity, education, and smoking [13]. Importantly, a bidirectional relationship exists between diabetes and periodontal disease [14]. In a recently published report, findings from a prospective study among rural Indian patients with type 2 diabetes demonstrated a positive linear correlation between periodontal disease and several diabetes characteristics, including an increased duration of diabetes and increased level of glycemic status defined by HbA1 levels [11]. The hypothesized mechanisms by which hyperglycemia could affect periodontal disease include the hyperinflammatory response to infection, the uncoupling of bone destruction and repair due to a more rapid collagen turnover, and the effects of advanced glycation end products [15]. The consistent findings for the associations between diabetes and periodontal disease in both directions in LMIC populations underscores the need for diabetic and dental screening and care in developing countries.

A classical study in the periodontal literature is based on a Sri-Lankan cohort back in the 1970s, describing “The natural history of periodontal disease in man” [16]. A Sri-Lankan tea worker group was established in 1970 and followed for a period of 15 years, showing that there are different patterns of disease progression in the absence of any professionally recommended or supervised oral hygiene practices. Based on tooth mortality rates, three subpopulations were identified: (1) individuals (approximately 8%) with rapid progression of periodontal disease (RP), those (approximately 81%) with moderate progression (MP), and a group (approximately 11%) who exhibited no progression (NP) of periodontal disease beyond gingivitis. The results indicate pathobiological patterns of periodontal disease progression independent of professional or individual oral care. 

The original cohort was re-examined 40 years later [17], and it was highlighted that patients with minimal periodontal attachment loss (<1.8 mm) at the age of 30 have an 80% chance of having approx. 20 teeth by the time they are 60 years old. With their long-term monitoring of this known Sri-Lankan cohort, the authors were able to reiterate that oral hygiene control is essential to preventing disease progression, further loss of attachment, and, ultimately, tooth loss.

Type 2 diabetes mellitus presents a substantial burden on the healthcare systems of developing countries. The prevalence of diabetes in Sri Lankan communities has increased significantly over the past decades. In the age-standardized reports, the prevalence of diabetes had increased from 2.5% in 1990 to 8.5% in 2000 [18], with increasing rates observed in both urban and rural communities [19]. The prevalence of overall, urban, and rural pre-diabetes was 11.5%, 13.6%, and 11.0%, respectively [4]. The projected diabetes prevalence for the year 2030 is 13.9%. These projections underscore the need to screen and identify diabetes complications.

Healthcare professionals with medical backgrounds lack awareness of the link between diabetes and dental outcomes [20]. While population data are not available in Sri Lanka, to our knowledge, the increased burden of diabetes suggests that the prevalence of periodontal disease may be prevalent. Despite the abundant evidence of the diabetic and dental relationship [7,21,22,23], still, medical professionals fail to inform patients with suggestions to seek dental checkups [24]. Practically, medical doctors are not used to incorporating questions about oral health into their clinical assessment [25]. Our findings suggest that the incorporation of dental assessment into diabetic screening and control, particularly in low- and middle-income countries, may be warranted. 

To our knowledge, this study is one of the few studies reporting on dental outcomes in a diabetic Sri Lankan population. However, this study has several limitations that should be noted. The first is the reliance on a binary single-item definition of dental outcomes, where items potentially lack specificity and could lead to under-reporting. It is recognized that this field-type study did not incorporate many traditional measures, such as periodontal probing, traditional charting, etc., or the specification of teeth, given that the field-testing was within a remote medical setting without access to dental facilities. Furthermore, the study is limited in its sample size as a pilot investigation. This study attempts to add data to the currently scant literature to demonstrate the overall impact of diabetes experience on the dental disease burden in LMICs. Further larger comprehensive studies that include validated formal dental assessments are warranted. 

## 5. Conclusions

In this study, we report an association between diabetes experience and dental risk in a Sri Lankan population. We found that poor glycemic control, indicated by increased capillary blood glucose levels, was associated with increased dental risk. These results can inform patients with type 2 diabetes, as well as their medical providers, of the importance of detecting and managing dental risk through lifestyle and medical interventions.

## Figures and Tables

**Table 1:** Distribution of dental outcomes and associated dental scores.

Dental Outcomes	Overall (*n* = 80)
Bleeding	16 (20.0%)
Recession	32 (40.0%)
Dental caries	47 (58.8%)
Abscess	2 (2.5%)
Mobile teeth	33 (41.3%)
Tooth fractures	39 (48.8%)
Missing teeth	56 (70.0%)
Suspicious oral mucosal lesions	3 (3.8%)
**Dental Score**	
0	14 (17.5%)
1	6 (7.5%)
2	14 (17.5%)
3	15 (18.8%)
4	14 (17.5%)
5	10 (12.5%)
6	6 (7.5%)
7	1 (1.3%)

**Table 2:** Demographic, clinical, and diabetes characteristics by the dental risk score.

Characteristics	Overall (*n* = 80)	Dental Risk Score 0–3 (*n* = 49)	Dental Risk Score 4–7 (*n* = 31)	*p*-Value *	Age-Adjusted OR (95% CI)
Age (years)	57 (50–64)			0.3	0.61 (0.25, 1.52)
≥57 (median)	40 (50%)	27 (68%)	13 (33%)		
<57	40 (50%)	22 (55%)	18 (45%)		
Gender				0.7	1.02 (0.31, 3.3)
Female	62 (78%)	37 (60%)	25 (40%)		
Male	18 (22%)	12 (67%)	6 (33%)		
Current Smoker					
Yes	1 (1%)	1 (100%)	0		
No	79 (99%)	48 (61%)	31 (29%)		
Body Mass Index (kg/m^2^)				0.4	
Obese (>30)	10 (13%)	8 (80%)	2 (20%)		0.26 (0.05, 1.43)
Overweight (25–30)	25 (31%)	16 (64%)	9 (36%)		0.67 (0.24, 1.88)
Normal (≤25) (ref.)	44 (55%)	25 (57%)	19 (43%)		
Systolic Blood Pressure (SBP) (mmHg)				0.6	0.92 (0.35, 2.39)
≥130	36 (45%)	23 (64%)	13 (36%)		
<130	44 (55%)	26 (59%)	18 (41%)		
Diastolic Blood Pressure (DBP) (mmHg)				0.2	0.60 (0.24, 1.50)
≥80	40 (50%)	27 (68%)	13 (33%)		
<80	40 (50%)	22 (55%)	18 (45%)		
Diabetes Duration (years)				0.8	1.61 (0.57, 4.54)
≥5	34 (43%)	20 (59%)	14 (41%)		
<5	46 (58%)	29 (63%)	17 (37%)		
HbA1C (%)				0.1	2.24 (0.64, 7.90)
>7	63 (79%)	36 (57%)	27 (43%)		
≤7	17 (21%)	13 (76%)	4 (24%)		
Diabetes Medication Use				0.8	1.18 (0.10, 13.8)
Yes	77 (96%)	47 (61%)	30 (39%)		
No	3 (4%)	2 (67%)	1 (33%)		
Capillary Blood Glucose (mg/dL)				0.03	2.93 (1.13, 7.61)
≥140	42 (53%)	21 (50%)	21 (50%)		
<140	38 (48%)	28 (74%)	10 (26%)		
Hypoglycemia				0.06	0.30 (0.10, 0.87)
Yes	28 (35%)	21 (75%)	7 (25%)		
No	52 (65%)	28 (54%)	24 (46%)		

Note: Totals may not = 100% due to missing data. * *p*-value based on the Jonckheere–Terpstra trend test for ordered variables (BMI), chi-square test if assumptions are met, Fisher’s exact test if the chi-square test assumptions are not met.

## Data Availability

Data are contained within the article.
